# A Review: Enhanced Anodes of Li/Na-Ion Batteries Based on Yolk–Shell Structured Nanomaterials

**DOI:** 10.1007/s40820-018-0194-4

**Published:** 2018-02-28

**Authors:** Cuo Wu, Xin Tong, Yuanfei Ai, De-Sheng Liu, Peng Yu, Jiang Wu, Zhiming M. Wang

**Affiliations:** 1 Institute of Fundamental and Frontier Sciences, University of Electronic Science and Technology of China, Chengdu, 610054 People’s Republic of China; 2 Department of Electronic and Electrical Engineering, University College London, Torrington Place, London, WC1E 7JE UK

**Keywords:** Yolk–shell structure, Lithium-ion batteries, Sodium-ion batteries

## Abstract

Lithium-ion batteries (LIBs) and sodium-ion batteries (SIBs) have received much attention in energy storage system. In particular, among the great efforts on enhancing the performance of LIBs and SIBs, yolk–shell (YS) structured materials have emerged as a promising strategy toward improving lithium and sodium storage. YS structures possess unique interior void space, large surface area and short diffusion distance, which can solve the problems of volume expansion and aggregation of anode materials, thus enhancing the performance of LIBs and SIBs. In this review, we present a brief overview of recent advances in the novel YS structures of spheres, polyhedrons and rods with controllable morphology and compositions. Enhanced electrochemical performance of LIBs and SIBs based on these novel YS structured anode materials was discussed in detail.

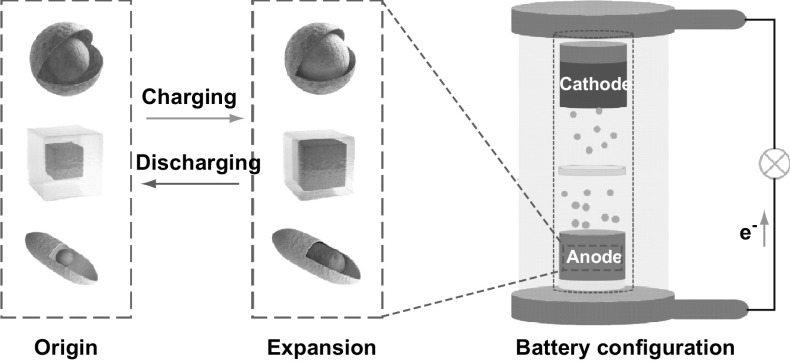

## Highlights

- In this review article, we have emphasized the recent developments of YS structured anodes and their applications for enhanced electrochemical performance in LIBs and SIBs.
- An overview of recent advances in the novel YS structures of spheres, polyhedron and rods with controllable shape and compositions is provided.
- Enhanced electrochemical performance of LIBs and SIBs based on these novel YS structured anode materials is discussed in detail.

## Introduction

Fossil fuels maintain the backbone for global energy supply. With the fast growth of energy consumption, it is urgent to exploit renewable energy [1]. Until now, solar and wind energy burst out a capability of relieving energy shortage [2]. Diverse energy conversion and storage devices are developed toward efficiently exerting solar and wind energy [3–7]. In this respect of energy storage from solar and wind energy to electrical energy, lithium-ion batteries (LIBs) account for a significant status due to its large capacity, long lifespan and high energy density [8, 9]. Furthermore, LIBs play a key role in the development of portable electronic devices and electric vehicles (e.g., laptops and cell phones) [10–13]. Generally, the configuration of LIBs consists of an anode, a cathode and electrolyte. During the process of charging and discharging, Li ions intercalate and deintercalate between two electrodes via the electrolyte [14]. The composition, morphology and structure of cathodes and anodes, together with the diffusion kinetics in the electrolyte, are significant and have been widely studied to thoroughly exert these superior properties in LIBs [8, 15, 16]. Particularly, the performance of LIBs significantly depends on the active anodes, which are used to store and release Li ions during charging and discharging possess [17, 18]. The most worldwide popular anode is graphite anode on account of its stable potential, low cost and long cycle life since its commercialization in LIBs by Sony Corporation [19, 20]. However, the graphite anode has a limited theoretical specific capacity of 372 mAh g^−1^ and poor rate capacity, which cannot satisfy the development of the portable electronic devices and electric vehicles [21–23]. Additionally, the global storage of lithium cannot afford massive application [24, 25]. Nevertheless, sodium enormously exists on earth [26, 27]. Therefore, sodium-ion batteries (SIBs) with working principle identical to LIBs are expected to be an alternative strategy for decreasing the cost of LIBs, even though the energy density of SIB is tentatively inferior to LIBs [25, 28, 29].

**Fig. 1:**
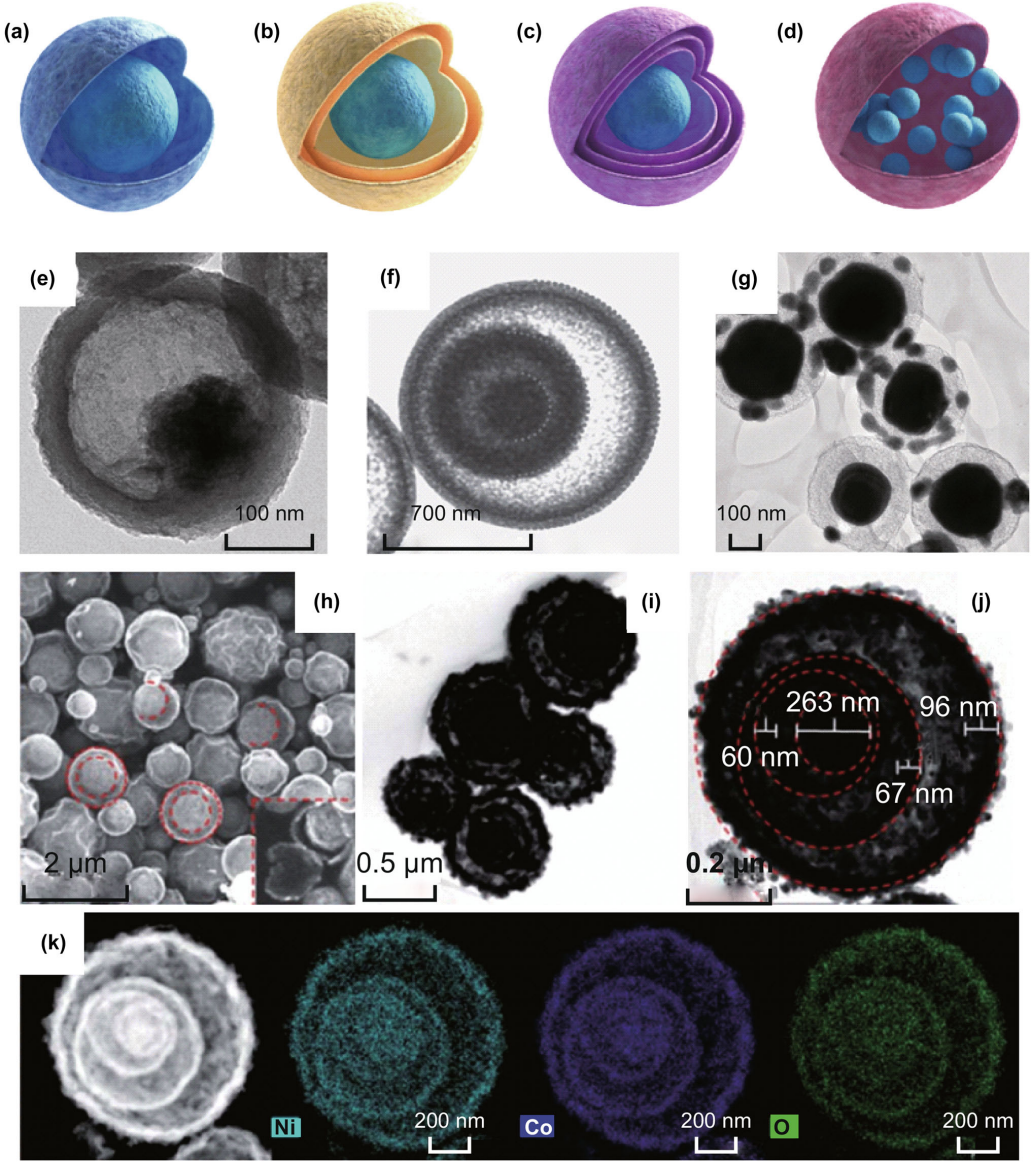
Graphical illustration of yolk–shell structure: **a** single shell with single yolk, **b** double shells with a single yolk, **c** multiple shells with a single yolk, and **d** single shell with multiple yolks. Reprinted with permission from Refs. [21, 55, 133]. TEM images of: **e** single shell with single yolk, **f** double shells with single yolk, and **g** single shell with multiple yolks. **h** SEM, **i** TEM, **j** HRTEM and **k** element mapping images of triple shells with single yolk. Reprinted with permission from Refs. [58, 60, 62, 68]

In order to boost the capacity of LIBs and SIBs, a number of novel structures have been studied [30, 31]. Recently, one kind of hollow structures [32–34], yolk–shell (YS) structures, has drawn much attention in applications of drug deliver [35], sensor [36], catalyst [37], LIBs and SIBs [38, 39]. Different from core/shell structure in dense contact [40], a typical spherical YS structure resembles frogspawn structure with a void interior, which provides movable space for core which can be also called yolk, as shown in Fig. 1a. Upon used as LIBs and SIBs anodes, YS structured materials are distinctive to improve electrochemical performance due to many advantages including unique buffering space, large surface area and short diffusion distance [41–44]. The void space of YS structured materials can address the problems of subversive volume expansion and avoid aggregation of electroactive cores during charging/discharging process. YS structured materials were first synthesized through silica template by Hyeon et al. [45]. Initial researches of YS structures concentrated on spherical structures [46–48]. Afterward, with the development of different synthetic methods such as selective etching [49], self-template [50], Ostwald-ripening [51, 52] and Kirkendall effect [46, 53], YS structures can be prepared into manifold types [54–56].

In this review article, we mainly emphasized the recent developments of YS structured anodes and their application for enhanced electrochemical performance in LIBs and SIBs. Following the introduction, in the second part, we provided a brief overview of typical and recent novel YS structures categorized into spheres, polyhedrons and rods. In the third and fourth part, we discussed these YS structure-designed materials toward the enhanced electrochemical performance in LIBs and SIBs, respectively. Subsequently, a summary was concluded in the final part.

## Development of Yolk–Shell Structures

### Sphere Structure

A typical spherical YS structure has a smooth spherical surface for both its shell and core. Generally, spherical YS structures vary in both shells and yolks, such as single shell with single yolk [57], double shells with single yolk [58, 59], multiple shells with single yolk [60, 61] and multiple yolks with single shell [62], which are shown in Fig. 1a–d. For a better understanding of these structures, we would review these structures based on different fabrication approaches. The YS structure can be fabricated not only from inside to outside but also outside to inside [49, 63–67]. As shown in Fig. 1e, the single shell with single core MoS_2_@C was synthesized by an etching strategy [68]. MoS_2_@PDA core–shell microspheres were transferred to single-shelled YS MoS_2_@C by annealing. Afterward, instead of calcination [69], the environmentally friendly H_2_O_2_ was used as an etching solution and tuned the void space with different concentrations between the MoS_2_ yolk and the carbon shell. However, using calcination method can synthesize complex structure. As depicted in Fig. 1f, the double-shelled YS SnO_2_@SnO_2_@SnO_2_ were fabricated through carbon calcination of three times. Carbon worked as hard template in designing this YS structure [70]. Polymerizing and carbonizing sucrose inside SnO_2_ particles generated the precursor of C–SnO_2_ composites. Due to lacking oxygen inside the dense core, the oxidation of carbon happens on the outside. The first combustion generated the C–SnO_2_/SnO_2_ core–shell. While under 1000 °C, the distinguishable thermal expansion coefficient of SnO_2_ and C–SnO_2_ resulted in the first void space. After another two times combustion and contraction, one yolk with double-shell structure was generated [58]. Using similar method, Leng et al. [60] accurately controlled the generation of triple-shelled NiCo_2_O_4_ spheres while polyvinylpyrrolidone served as a template. Figure 1h, i proves that the as-prepared multi-shell spheres had uniform sizes. Ni, Co and O elements were detected and evenly distributed in dot-mapping images of Fig. 1k. The four red dashed cycles in Fig. 1j show the morphology of three shells and one yolk. Apart from a single yolk, multiple yolks with a single shell Sn_4_P_3_@C were fabricated from outside single carbon shell to inside multi-yolks. The transmission electron microscopy (TEM) image (Fig. 1g) shows the chief yolk abounded with tiny yolks [62].

**Fig. 2:**
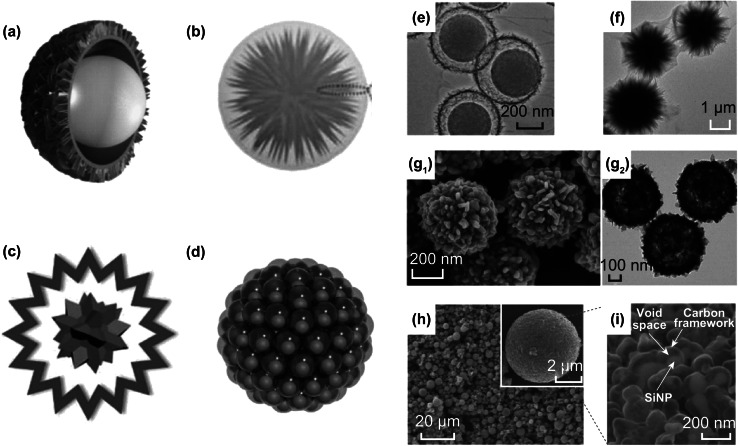
Schematic demonstration of novel YS structure of: **a** polystyrene spheres (PS)@NiCo_2_S_4_. Reprinted with permission from Ref. [71]; **b** urchin-like Bi_2_S_3_@polypyrrole(ppy). Reprinted with permission from Ref. [72]; **c** rough VO_2_ microspheres. Reprinted with permission from Ref. [73]; and **d** pomegranate-like Si@C. Reprinted with permission from Ref. [75]. The corresponding TEM images of **e** PS@NiCo_2_S_4_, and **f** Bi_2_S_3_@ppy. Reprinted with permission from Ref. [71, 72]. **g**_**1**_ FESEM and **g**_**2**_ TEM images of VO_2_ microspheres. Reprinted with permission from Ref. [73]. **h** SEM image of pomegranate-like Si@C particles. Inset shows the spherical morphology in overall. **i** Details of sub-YS nanospheres in a single pomegranate-like Si@C particle. Reprinted with permission from Ref. [75]

Recently, a number of novel YS spheres have emerged. Different with conventional YS spheres with smooth shell and yolk, the shell or the yolk of the novel YS spheres possesses various surfaces. The coconut-like polystyrene (PS)@NiCo_2_S_4_ YS sphere (shown in Fig. 2a) was synthesized from inside to outside by Zhu et al. [71]. Silicon dioxide was used for template and then removed during the hydrothermal process. Figure 2e exhibits numerous nanosheets around the shell which endow ultrahigh surface area exceeding 200 m^2^ g^−1^ and penetrable property of this YS structure. In addition to changing shell morphology, the yolk can also be transformed. Liang et al. [72] were inspired by cirsium flower and assembled Bi_2_S_3_ nanowires to fabricate a yolk of radial pattern which was coated by polypyrrole (PPy). On account of the whippy PPy, the designed architecture possesses mechanical flexibility, short diffusion distance and large surface area. In Fig. 2f, we can see that the flexible shells are expanded by inside radial Bi_2_S_3_ nanowires. Furthermore, Ma et al. [73] tried to synthesize rough shell and rough yolk spheres (Fig. 2c) from outside to inside through a template-free method. In this template-free method based on Ostwald-ripening mechanism [74], dissolution and recrystallization formed the VO_2_ YS spheres. The scanning electron microscope (SEM) image in Fig. 2g_1_ and TEM image in Fig. 2g_2_ clearly demonstrate the rough and high surface area and the rough yolk inside. In addition to independent single YS sphere, Cui et al. developed a single main sphere consisting of many sub-YS spheres. Figure 2d shows multiple sub-YS architectures of Si@C that is inspired by pomegranate [75]. In the fabrication process, silicon dioxide was etched to engender void space.

### Polyhedron Structure

**Fig. 3:**
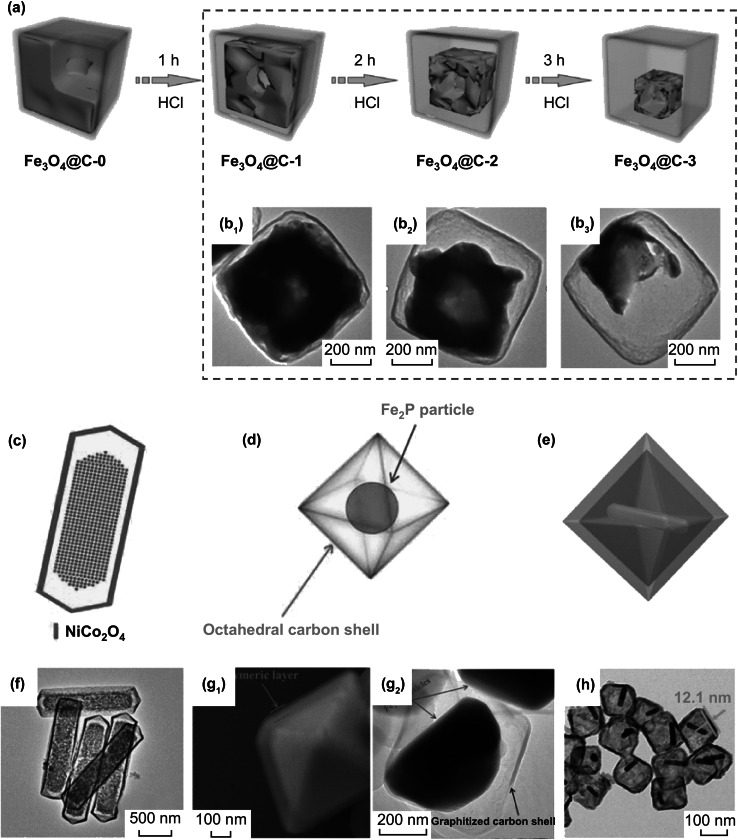
**a** Schematic illustration YS Fe_3_O_4_@C nanobox following 1-, 2- and 3-h etching time. The TEM images (**b**_**1**_, **b**_**2**_, **b**_**3**_) of Fe_3_O_4_@C nanobox are shown in the dashed box corresponding to 1-, 2- and 3-h etching, respectively. Reprinted with permission from Ref. [84]. Graphical illustrations of **c** YS nanoprism of Ni–Co mixed oxide, **d** YS octahedral Fe_2_P@C, and **e** YS octahedral Au nanorod@Cu_7_S_4_. **f** TEM image of YS Ni–Co mixed oxide nanoprism. **g**_**1**_ SEM image and **g**_**2**_ TEM octahedral Fe_2_P@C. **h** TEM octahedral Cu_7_S_4_ shell with Au nanorod yolk. Reprinted with permission from Refs. [80, 82, 88]

The sphere is not the only YS architectures. YS polyhedron has also drawn much attention due to its tailored shape with more surfaces in energy conversion [76–79]. YS polyhedron can be categorized in pentahedron [80], hexahedron [81], octahedron [82] and dodecahedron [83]. Fe_3_O_4_@C YS nanobox of hexahedron was successfully synthesized by Liu et al. [84]. Polydopamine (PDA) wrapped the Fe_2_O_3_ nanocubes which had the size of 530 nm. After calcination, Fe_2_O_3_@PDA became Fe_3_O_4_@C and engendered interior void space between yolk and shell due to Ostwald-ripening effect. The void space expanded after etching. Figure 3a illustrates the schematic transformation of the Fe_3_O_4_ yolk with different etching reaction time by hydrochloric acid (HCl) solution. Figure 3b_1_, b_2_ shows TEM images of Fe_3_O_4_@C YS nanobox after 1-, 2- and 3-h etching, respectively. The thickness of the carbon shell was 20 nm. With increasing time, the Fe_3_O_4_ yolk decreased from initial 530 nm to 470, 380, and 230 nm. Using similar method, He et al. [85] also fabricated the sulfured Fe_3_O_4_@C YS nanobox via the extra melt-diffusion process. Meanwhile, Liu et al. [86] sulfured Fe_3_O_4_@C to FeS_2_@C with sulfur powder through combustion. Besides, Su et al. [81] utilized metal–organic frameworks (MOFs) to drive the generation of CdS microboxes based on the anion exchange and Kirkendall effect process. Excepting for tuning yolk size through etching, the thickness of shell can be controlled. Varying the PDA concentration is used to successfully synthesize different carbon shells with sizes of 15, 25, and 35 nm in Sn@C nanoboxes by Zhang et al. [87]. Unlike nanobox with uniform six surfaces, FeO_*x*_@C YS hexahedron with two rhombus and other unequal surfaces was proposed by Yu et al. [78]. Interestingly, a prism structure of Ni–Co precursor was contracted to Ni–Co oxide YS nanoprism (as depicted in Fig. 3c) via thermal treatment in air [80]. Heterogeneous contraction happened from vertical and lateral directions in the prism structure. Figure 3f demonstrates the prism-like shell and yolk. During the contraction, two competitive forces of contraction force and adhesive force coexisted. Ni–Co precursor core inward contracting was caused by the contraction force. On the contrary, the adhesive force outward expanded the core due to carbon dioxide releasing from organic decomposition. Based on hydrothermal reaction, the octahedral structure of Fe_2_PO_5_/polymer serving as precursor and template was synthesized via one-step method. After calcination, dense octahedral structure transformed into hollow octahedral graphitized carbon (GC) shell with Fe_2_P yolk, as shown in Fig. 3d. SEM image of Fig. 3g_1_ and TEM image of Fig. 3g_2_ demonstrate the YS octahedral structure. Another Au@Cu_7_S_4_ YS octahedral structure with nanorod yolk is shown in Fig. 3e [88]. The nanorods can be clearly found in Fig. 3f.

### One- and Three-Dimensional Structures

**Fig. 4:**
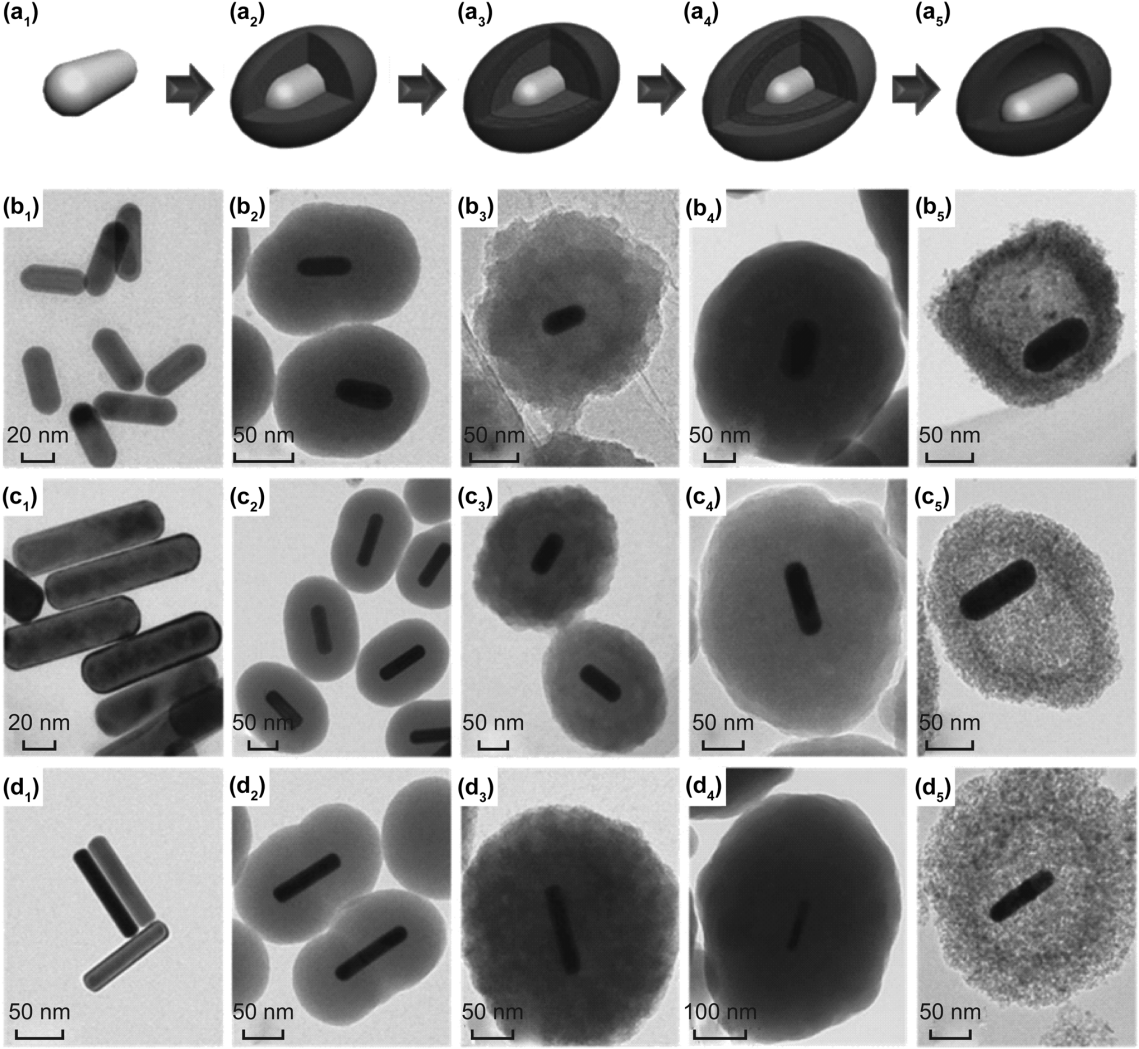
**a**_1_–**a**_5_ Schematic illustration of synthesis process of YS Au@TiO_2_ nanorod. The corresponding TEM images of synthesis process: **b–d** the as-prepared Au@TiO_2_ nanorod with different aspect ratios. Reprinted with permission from Ref. [92]

Additionally, one-dimensional (1D) YS rods can offer wider lateral void space and shorter ion diffusion length. Combining nanorod yolk in YS structure, rod-like YS structures with rod shell are also studied [89–91]. Li et al. [92] prepared an Au@TiO_2_ YS nanorod structure. By using decyltrimethylammonium bromide (CTAB), absorption preference of high-energy crystal planes on gold seeds generated the Au core nanorod structure. As can be seen in Fig. 4a_1_–d_1_, the gold seeds further grew along {111} planes which are not wrapped by CTAB. With diverse Au nanorod aspect ratios, SiO_2_ can work as a template to form hollow space, as shown in Fig. 4a_2_–d_2_. TiO_2_ was easily broken down during calcination. In order to fabricate stable TiO_2_ shell, as depicted in Fig. 4a_3_–d_3_, the SiO_2_ shell was secondly coated as protective shell, as shown in Fig. 4a_4_–d_4_. SiO_2_ packing agent is tetraethyl orthosilicate (TEOS) and TiO_2_ packing agent is tetrabutyl titanate (TBOT). After calcination and etching, the YS rod shell and rod yolk structure was formed, which are depicted in Fig. 4a_5_–d_5_. Different with above rod in rod YS structure, Zhang et al. [93] demonstrated a Fe_3_O_4_@Fe_3_C@C YS structure of nanorod (nanospindle) shell with spherical yolk. The nanospindle carbon shell with thickness of 3–5 nm was formed via using *α*-Fe_2_O_3_ nanospindle as precursor coated with resorcinol formaldehyde (RF). After carbonization, the core–shell Fe_3_O_4_@Fe_3_C acting as the yolk possessed a size of 15–20 nm. The interior void space largely occupied 75% of total inside volume. Xu et al. [94] designed a tree branch YS structure. Multi-walled carbon nanotube (MWNT) served as trunk to support the rod branches of YS Fe_2_O_3_@C. Coating the template of SiO_2_ was for interior hollow space generation in etching step. Another self-templated Sn@SnO_*x*_ YS nanosphere structure arrayed in nanofiber was proposed by Kang et al. for the first time [64]. The carbon nanofiber with Sn nanospheres was produced by electrospinning. The Sn nanospheres densely arrayed along the C nanofiber. During calcination, C nanofiber was decomposed. Besides, the Sn nanospheres were oxidized to form SnO_*x*_ shell and contracted to form Sn yolk due to Kirkendall diffusion effect.

### Advanced Effects in the Structures

Hollow structures are indeed a strategy to facilitate electrochemical performance of LIBs and SIBs attributing to the void cavity and thin shell. However, volume energy density is decreased along with the formation of hollow structures. YS structures possessing a suitable void space between yolk and shell can accommodate the volume expansion of yolk to maintain a sufficient volume energy density. The expansion ratio of yolk depends on the intrinsic properties of yolk materials. The well-defined void space can accommodate lithium/sodium [95]. In contrast, the extra void space will decrease the volume energy density. Additionally, a large contact area between yolk and shell promotes electron and ion diffusion [96]. Therefore, engineering the void space and large contact area is a key factor to tune the electrochemical performance. The three types of YS structures mentioned above have different effects on void space and contact area. The final morphologies of sphere, polyhedron and rod YS structures mostly originate from initial shapes of yolks. The precursors are previously fabricated in forms of spherical shape, cubic shape, octahedral shape and rod shape. Compared to typical YS spheres, the yolks in polyhedron shell have a larger contact area, which can facilitate conducting electrons and diffusing ions [87]. However, the polyhedron shells suffer from a larger tension on the surface. The yolks in the rod structures can expand along the length direction which have a very large void space size. Hence, the rod structures can be used for yolk materials with a large expansion ratio. Novel strategies like multiple yolks and densely stacking nanostructures cannot be scalably used. It is still noteworthy to exploring controllable and stable YS nanostructures.

## Performance of Yolk–Shell Materials in Lithium Batteries

**Fig. 5:**
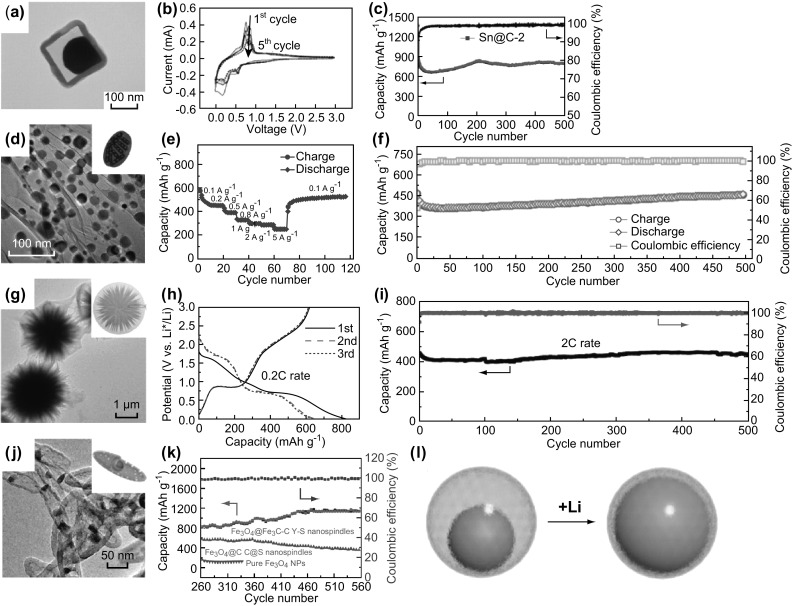
TEM images and electrochemical performance of LIB assembled with YS structure anode: **a** Sn@C nanocube, the corresponding **b** cyclic voltammetry curves and **c** cyclic performance. Reprinted with permission from Ref. [87]. **d** Ni_2_P wrapped by graphene networks, the corresponding **e** rate performance at different current densities, and **f** long-term cycle performance. Reprinted with permission from Ref. [124]. **g** urchin-like Bi_2_S_3_@ppy, the corresponding **h** galvanostatic profiles and **i** long-term cyclic performance. Reprinted with permission from Ref. [72]. **j** Fe_3_O_4_@Fe_3_C nanospindle and **k** the corresponding long-term cyclic performance. Reprinted with permission from Ref. [93]. **l** Schematic illustration of volume expansion of YS structure yolk after lithiation. Reprinted with permission from Ref. [104]

The YS structure with unique buffering space, large surface area and short diffusion distance shows great superiority as next-generation LIBs anodes [97–99]. Silicon is one of the promising materials for LIBs anodes [100–103]. Cui et al. [104] fabricated a YS Si@C spherical structure at room temperature. The Si assembled LIB performed a high capacity of 2800 mAh g^−1^ at 0.1 C. After 1000 cycles, it still held 74% capacity retention and 99.84% Coulombic efficiency. During the charging and discharging processes, the void space allowed the electroactive yolk to expand freely and avoided the yolks aggregating with other yolks. Figure 5l illustrates the expanded state of the YS structure after intercalating Li ions. The carbon shell worked as a framework to support the entire structure and avoid cracking. The hollow carbon shell initially has nanopores. After several cycles, solid-electrolyte interphase (SEI) is formed on the surface of the carbon shell to separate the electrolyte and Si yolk. Another YS pomegranate-like Si@C obtained a capacity of 2350 mAh g^−1^ at 0.05 C was also proposed by Cui et al. [75]. Its volume capacity of 1270 mAh cm^−3^ was twice higher than graphite anode. The capacity retention remained 97% during the second 1000th cycle at 0.5 C. This densely packed structure resulted in a larger contact area of electrode–electrolyte and formation of a thin and stable SEI film, which led to high Coulombic efficiency of 99.87%. Its high Coulombic efficiency indicated the well reversible cycles of the electrode. Moreover, insufficient void space in sub-particles of this pomegranate microparticle resulted in cracks, which made SEI excessively form. Therefore, internal void space should be critically controlled. Similarly, Han et al. [105] coated Fe_3_O_4_ sub-nanoparticles with the pomegranate-like carbon shell, which resulted in an excellent specific capacity of 1246 mAh g^−1^ at 0.8 A g^−1^ and extremely impeded the decrease of volume energy density. Integrating design is an attractive strategy for LIBs. Liu et al. [106] achieved a silicon@silica@void@carbon YS nanosphere structure and interlinked these nanospheres through chemical bonding with carboxymethyl cellulose and citric acid polymer binder, which performed a high specific capacity of 1640 mAh g^−1^ at 1 A g^−1^ and excellent stability that maintained 1000 mAh g^−1^ at 5 A g^−1^ after 1000 cycles. Except that silicon suffers the problem of volume expansion, SnO_2_ with a high theoretical capacity of 790 mAh·g^−1^ similarly faced this problem [107]. Wang et al. [107] synthesized SnO_2_@C YS nanosphere with thin carbon shell of 15–25 nm and tailored the large interior hollow space of 100–160 nm. This SnO_2_@C showed the high capacity of 2190 mAh g^−1^ in the first cycle. Also, Choi et al. [108] synthesized a YS double-shell SnS spheres which can deliver a specific capacity of 672 mAh g^−1^ for 150 cycles at 1 A g^−1^. The Coulombic efficiency was stably maintained at 99% in the posterior cycles. Fan et al. [109] coated ZnO yolk with carbon shell. The resultant YS ZnO@C performed an ultra-stable cyclic performance of 5000 cycles at a current density of 10 A g^−1^ and kept 96.9% retention rate. Unlike popular carbon shell [110–112], Pan et al. [113] synthesized a ZnO/NiO shell composed of plentiful nanorods to encapsulate yolk materials. The composed nanorods on shell can promote electrolyte penetrating, and the ZnO/NiO YS spheres performed a high specific capacity of 1008.6 mAh g^−1^. Li et al. [114] synthesized YS composites of Al yolk wrapped by the thin TiO_2_ shell with a thickness of 3 nm. TiO_2_ cannot compete with Al on specific capacity. The thick TiO_2_ is adverse to electron and ions transport. According to the model calculation, TiO_2_ should be as thin as 10 nm or less. Generally, a thin shell cannot take too much internal tension. Fortunately, nanosized TiO_2_ was thinned to a few nanometers while had sufficient mechanical strength. This Al@TiO_2_ YS nanosphere possessed a high rate of 1200 and 650 mAh g^−1^ at 1 C and 10 C, respectively, after the same 500 cycles and realized a robust 99.2% Coulombic efficiency at 1 C. Kim et al. [115] filled structurally stable nitrogen-doped graphitic carbon in YS FeO_*x*_ sphere. It showed the excellent capacity performance of 1071 mAh g^−1^ for 1000 cycles and high rate capacity of 598 mAh g^−1^ at 10 A g^−1^. The as-prepared YS metal oxide microsphere was further connected to the yolk and shell with porous nitrogen-doped graphitic carbon (NGC). The NGC not only possessed high electric conductivity but also provided structurally supporting, which offseted the volume energy density caused by void space. Spray pyrolysis is a highly scalable method for producing electrode materials, which can fabricate plenty of specific structured materials such as alloys, oxides, nitrides and organic–inorganic composites [116–119]. Choi and coworkers [120] used spray pyrolysis method to generate a YS structured with 7-10 components. During 100 cycles at 1 Ah g^−1^, 7, 8, 9, and 10 components performed 735, 647, 712, and 543 mAh g^−1^, respectively. Among these four specimens, 8 components increased to 1015 mAh g^−1^ from 300 to 1000 cycles and sustained a Coulombic efficiency of 99.8% during cycle.

As mentioned above, the typical spherical YS structure is not the only strategy of designed YS structure for enhancing electrochemical performance of LIBs. Zhang et al. [87] realized a YS Sn@C nanobox with adjustable shells of 15–35 nm to tune the void space. As exhibited in Fig. 5a, the Sn spherical yolk closely pasting on the nanobox C shell provided void space and large contact between yolk and shell. The melting point of Sn is as low as 232 °C. Thermal calcination leads to liquid state Sn in the box-shaped shell. After cooling, the liquid state Sn turned to the solid state which naturally contacted the inner surface of cubic carbon shell. The natural contact provided higher transport pathway of electrons and ions due to larger contact area. The adequate void space can satisfy the volume expansion. The Coulombic efficiency only reached 61% caused by the initial formation of SEI, while in the back cycles, the Coulombic efficiency was obtained at 98–100%. Figure 5b shows the form of Li_x_Sn alloy in cyclic voltage peak. Figure 5c demonstrates the optimized shell of 25 nm performing, resulting in a stable capacity of 810 mAh g^−1^, even after 500 cycles. In addition to Sn@C, octahedral YS CuO@C with multiple yolks was designed by Tao et al., which exhibited the performance of 512 mAh g^−1^ at 500 mA g^−1^ after 300 cycles. Compared to single yolk, the multiple yolks have more contact area which can accelerate electron and ion diffusion [121]. Yu et al. [122, 123] put forward a three-dimensional (3D) YS structure of Ni_2_P nanoparticles wrapped by porous graphene networks, since graphene possesses high conductivity, as shown in Fig. 5d [124]. The porous graphene possessed high surface area. The Ni_2_P nanoparticles were formed by annealing reaction of NiNH_4_PO_4_·H_2_O nanorods. The NiNH_4_PO_4_·H_2_O nanorod served as a self-assemble template for encapsulation of graphene and engendering void space. Remarkably, this 3D Ni_2_P@graphene (Ni_2_P@pG) structure showed excellent rate performance under different current density (Fig. 5e). Moreover, even at a large current density of 0.3 A g^−1^, its capacity possessed 457 mAh g^−1^ during 500 cycles of a long-term test in Fig. 5f. Its Coulombic efficiency was obtained at 99%. Mo et al. also used 3D graphene to realize a complex YS structure [125]. They deposited N-doped graphene on Ni foam firstly. The GeO_2_@Ni were loaded on 3D N-doped graphene afterward. Then the 3D N-doped graphene was deposited again. After reduction and etching, the 3D Ge quantum dot@N-doped graphene YS nanostructure was formed. This YS architecture not only rapidly conducted ions and electrons through high pathway porous graphene foam, but also preserved necessary tiny void space on distributed Ge quantum dots, which extremely relieved the decrease in volume energy density. The final as-prepared YS nanostructure performed 1220 mAh g^−1^ for 1000 cycles and ultrahigh rate capacity of 800 mAh g^−1^ at 40 C. Transforming the yolk shape can also give rise to large surface area and short diffusion distance [126, 127]. Liang et al. [72] designed an urchin-like Bi_2_S_3_ yolk assembled from nanowires. Figure 5g shows flexible shell film and urchin-like yolk. Compared to typical single spherical yolk, the urchin-like yolk has more surface area and contact sites linking to shell, which can guarantee high ions transport. Meanwhile, the branches of urchin yolk guarantee the volume expansion in the limited void space. As depicted in Fig. 5h, the initial charging and discharging capacities were 824 and 621 mAh g^−1^, respectively. Besides, the YS structure with urchin-like yolk had a stable performance (Coulombic efficiency of 95% over 500 cycles in Fig. 5i) at a high rate of 2 C. After the long-term cycle, it insisted a relatively high capacity of 450 mAh g^−1^. As mentioned before, the YS Fe_3_O_4_@Fe_3_C–C nanospindle with ultra-large void space of 75% volume ratio can buffer larger volume expansion [93]. This nanospindle showed a great reversible capacity of 1128.3 mAh g^−1^ and kept 1120.2 mAh g^−1^ for 100 cycles at 500 mA g^−1^. Figure 5j, k shows the electrochemical performance of YS nanospindle-based LIB.

## Performance of Yolk–Shell Materials in Sodium Batteries

YS structured anodes not only can enhance LIBs performance, but also can improve sodium storage. Considering the abundant storage of sodium, SIBs also have great potential in practical applications [128]. It is significant to explore the ways to enhance the performance of SIBs [129]. In a relevant work, by synthesized YS Sn_4_P_3_@C nanospheres, Yu’s group solved the problem of huge volume expansion of sodium alloy that would damage the anode of SIB [62]. As shown in Fig. 1g, the Sn_4_P_3_@C nanospheres had multiple yolks. The multiple yolks have advantage of large contact areas than single yolk to conduct electrons and ions. The Sn_4_P_3_@C anode integrated SIB exhibited a high capacity of 790 mAh g^−1^ and robust cyclic performance of 400 cycles at 1.5 C with high Coulombic efficiency. Li et al. [111] connected single YS Sn@C nanospheres through an eggette-like carbon membrane structure. As SIB anode, it could hinder nanospheres aggregation and provide fast pathways for Na^+^ transportation, which resulted in a capacity of 400 mAh g^−1^ at 0.1 C. The membrane carbon shell restrained the aggregation of Sn nanoparticles and connected all the YS Sn@C nanoparticles through itself to generate highway of ions and electrons. TiO_2_ definitely limits the storage of Na due to its confined conductivity of electrons and diffusion of ions. Even though the TiO_2_ as SIB anode material showed relatively low capacity, Zhang et al. [130] hence took YS nanospheres into account for enhancing TiO_2_ storage ability of sodium. They synthesized the N-doped and carbon tuning YS TiO_2_ via Ostwald-ripening effect. The designed YS nanostructure with the large amount of nanosheets on its shell increased the inserted sites of Na ions due to large surface area. Therefore, these nanosheets provided a shortcut for ions diffusion. Moreover, the N-doped carbon decreased the band gap for fast movement of Na ions toward TiO_2_. Because of these synergetic effects, it showed a specific capacity of 242.7 mAh g^−1^ at 0.5 C. With current density increased to 1 C, it maintained 200.7 mAh g^−1^ during 550 cycles and almost 99.8% Coulombic efficiency. Moreover, its excellent performance of capacity retention kept at 95.5% for 3000 cycles in harsh condition of 25 C. Afterward, Qiu et al. [131] implemented a more severe test on YS TiO_2_@C. It obtained a capacity of 210 mAh g^−1^ with the stable Coulombic efficiency at 99.5% and stuck to 80% retention capacity of 2000 cycles at 40 C. Sb is an alternative yolk material for SIB. Liu et al. [132] use Sb as yolk and carbon as shell to obtain a specific capacity of 280 mAh g^−1^ at 1 A g^−1^. Given a highspeed diffusion pathway along *c*-axis MoS_2_, Geng et al. [133] synthesized MoS_2_ shell outside Co_9_S_8_ yolk as SIB anode. The Co_9_S_8_@MoS_2_ spheres showed a notable cyclic performance of 300 mAh g^−1^ for 1200 cycles at 2 A g^−1^.

**Fig. 6:**
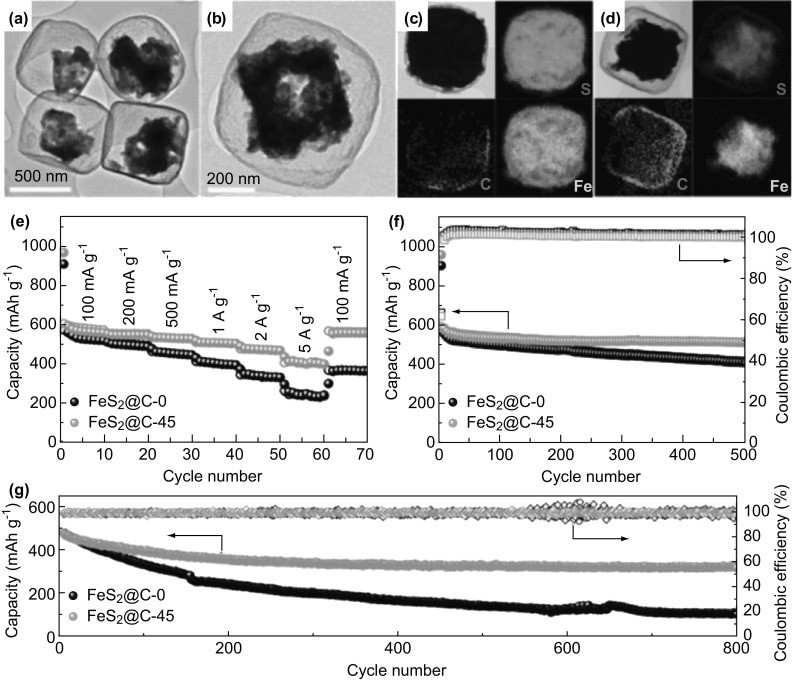
**a** TEM image and **b** HRTEM image of FeS_2_@C with 45-minute etching. Element mapping images of: **c** FeS_2_@C-0 and **d** FeS_2_@C-45. **e** Comparison of rate performance of FeS_2_@C-0 and FeS_2_@C-45 at various current densities. **f** Variation of capacity retention of FeS_2_@C-0 and FeS_2_@C-45 in 100 cycles performance at 100 mA g^−1^. **g** Long-term cyclic performance of FeS_2_@C-0 and FeS_2_@C-45. Reprinted with permission from Ref. [86]

FeS_2_ with a theoretical capacity of 894 mAh g^−1^ drew Liu et al. attention [86]. They synthesized a YS structure of nanobox. Figure 6a, b demonstrates the TEM and HRTEM images of FeS_2_@C nanobox with 45-min etching, which clearly shows the nanobox shape with a yolk. The element mapping images of FeS_2_@C nanobox without etching (FeS_2_@C-0) and with 45-min etching (FeS_2_@C-45) are shown in Fig. 6c, d. The element mapping images proved that Fe, S and C were homogeneously distributed in the YS nanobox. Particularly, Fig. 6e–g compares the two FeS_2_@C-0 and FeS_2_@C-45 anodes. The FeS_2_@C-45 revealed the more stable variation of specific capacity than FeS_2_@C-0 at different current densities during long cyclic test. After 100 cycles, the FeS_2_@C-45 performed a capacity of 511 mAh g^−1^ at 100 mA g^−1^. After further 800 cycles, the FeS_2_@C-45 still obtained 330 mAh g^−1^ at 2 A g^−1^ with around full Coulombic efficiency. Another YS Sn_3_P_4_@C nanobox structure was generated by Ma et al. [134]. They fabricated ZnSn(OH)_*x*_ nanobox firstly and used it as a template then to generate Sn_3_P_4_@C nanobox. As SIB anode, the YS Sn_3_P_4_@C nanobox relieved Sn_3_P_4_ yolk expansion and showed the remarkable cyclic performance of 516 mAh g^−1^ at 1 A g^−1^ for 500 cycles with the Coulombic efficiency of 99.0%. Additionally, YS Sb@Ti–O–P nanorods were synthesized for SIB anode to reach a high capacity of 760 mAh g^−1^ at 500 mA g^−1^ after 200 cycles [90]. Another novel YS dodecahedron structure of Co_3_O_4_@C for SIB anode showed a capacity of 307 and 269 mAh g^−1^ at 1000 and 2000 mA g^−1^, respectively [77].

As a result of the enhanced performance of YS materials applied in LIBs and SIBs, it can mainly attribute to the functional YS shell. Due to the hollow shell, the yolk can be relatively increased weight fraction to increase energy density compared to the dense yolk–shell structure. More than that, the hollow shell provides void space to buffer yolk expansion and avoid aggregation of yolks. The shell is thin enough and has a large surface area which can highly lower diffusion distance. The spherical YS type is superior in low specific area which can conduce to reducing surface tension. The void size in typical spherical spheres can be further filled with porous NGC to facilitate electrons diffusion and decreased volume energy density. The densely stacked spherical nanoparticles can efficiently relieve the decrease of volume energy density [135]. However, the spherical spheres cannot have enough contact area between yolk and shell for electrons conducting and ions diffusing. The polyhedron can provide more surface for yolk contacting instead. In addition, the rod YS structures possess high aspect ratio. This structure can be used for large expansion ratio-based yolk materials. Carbon is mostly used as shell materials. However, the amorphous carbon shell with large sites can trap lithium, which will resulted in decreasing Coulombic efficiency. Si-based anode have large capacity, while using porous Si as yolk material will partially release the stress of volume expansion [136–138]. Overall, the electrochemical performance of the YS structured anodes has been effectively improved in LIBs and SIBs.

## Conclusion and Perspectives

The YS structures in LIBs and SIBs are reviewed in this paper. Typical spherical YS structures are tuned with various numbers of shells and yolks varying in number and size. To explore more spherical YS structures, the rough shells were formed with large surface area. The yolks are changed in self-assembled nanowires. Also, sub-YS nanospheres were gathered to form a pomegranate-like nanosphere. Interestingly, a box-shaped shell with box yolk YS structure is synthesized via Ostwald-ripening effect. Other than box-in-box structure, prism and octahedron shells with nanoparticle and nanorod and rod-shaped YS structures are also discussed. The YS structured materials with tailored interior space, shape and components can synergetically relieve volume expansion and facilitate rapid diffusion and electron transportation. Due to these superior merits, the YS structured anodes immensely improved Li and Na storage performance, which resulted in high specific capacity, excellent rate capability and stable long-term cyclic performance. These YS structured materials indeed showed promising performance as LIBs and SIBs anodes. Partial results are concluded in Table 1.

**Table 1:** Electrochemical performance of YS structured materials

Shape	Battery	Composition	Capacity (mAh g^−1^)	Cycle numbers	Current density (A g^−1^)	References
*Sphere*						
Single shell	LIB	Si@C	1500	1000	0.1 C	[104]
Double shells	LIB	SnO_2_@SnO_2_@ SnO_2_	704	40	0.625	[58]
Pomegranate	LIB	Si@C	2350	1000	0.05 C	[75]
Single shell	LIB	SnO_2_@C	630	100	0.1	[107]
Double shells	LIB	SnS	672	150	1	[108]
Single shell	LIB	FeO_*x*_/N-doped GC	1071	1000	1	[115]
Single shell	LIB	Al@TiO_2_	650	500	10 C	[114]
Single shell	LIB	ZnO/Ni3ZnC0.7/C	1002	750	0.5	[139]
Single shell	LIB	ZnO@C	659	300	0.5	[109]
Triple shells	LIB	Ni–Co oxide	1064	100	0.4	[60]
Urchin yolk	LIB	Bi_2_S_3_@PPy	337	500	10 C	[72]
Eggette-like	SIB	Sn@C	200	1000	1	[111]
Single shell	SIB	TiO_2_@C	136	2000	1 C	[131]
Multiple yolks	SIB	Sn_4_P_3_@C	360	400	1.5 C	[62]
Single shell	SIB	N-doped TiO_2_	200.7	550	1 C	[130]
*Polyhedron*						
Prism	LIB	Ni–Co oxide	1028.5	30	0.2	[80]
Cube	LIB	Fe_3_O_4_@C	475	8000	10	[84]
Cube	SIB	FeS_2_@C	330	800	2	[86]
Cube	LIB	Sn@C	810	500	0.2	[87]
Cube	SIB	Sn_3_P_4_@C	516	500	1	[134]
Hexahedron	LIB	FeO_*x*_@C	810	100	0.2 C	[78]
Octahedra	LIB	CuO@C	512	300	0.5	[121]
Octahedra	LIB	Fe_2_P@GC	592	200	0.1	[82]
Dodecahedron	LIB	Co_3_O_4_@C	1100	120	0.2	[77]
*1D and 3D*						
Rod	SIB	Sb@Ti–O − P	760	200	0.5	[90]
Spindle	LIB	Fe_3_O_4_@Fe_3_C	1120.2	100	0.5	[93]
Tree branch	SIB	Fe_2_O_3_@C on MWNT	272	100	0.16	[94]
Tree branch	LIB	Fe_2_O_3_@C on MWNT	1024	360	0.2	[94]
3D porous	LIB	Ge@N-doped graphene	1220	1000	1 C	[125]
Rod	LIB	Ni_2_P@pG	457	500	0.3	[124]

In order to more precisely control the shape and size, further study should be focused on exploring accessible and advanced approaches. Rigid materials such as silicon and silica usually act as sacrificial layers to engineer the internal void space between the yolk and shell. However, the dissolution solvents, like hydrofluoric acid that can etch the sacrificial layers, are harmful. The environmentally friendly etching solutions should be explored. The densely stacked nanoparticles provide a strategy to sustain the volume energy density. The advanced methods which can stack polyhedron/rod YS structures and increase contact areas are significant in the future developments. In addition, morphology control would not be confined to these three types. It also appears a trend to explore two-dimensional YS nanosheets. The alternative materials with even lower cost should be explored for scalable and commercial usage. Carbon is still the most popular yolk–shell materials with good electric conductivity. Future works should concentrate on designing the void space size according to the expansion fraction of yolk materials. Na ion is bigger than Li ion, which results in a larger volume expansion in the YS structure. It’s also necessary to find materials with a small expansion ratio of Na ions and high theoretical specific capacity. Undoubtedly, novel YS structures with tailored and functional components are crucially promising to enhance LIBs and SIBs performance in the near future with commercially available and industrially scalable application.
